# The Effects of Combined Versus Single-Mode Resistance and Repeated Sprint Training on Physical Fitness, Hematological Parameters, and Plasma Volume Variations in Highly Trained Soccer Players

**DOI:** 10.3390/sports12110290

**Published:** 2024-10-24

**Authors:** Abderraouf Ben Abderrahman, Ali Aloui, Nidhal Jebabli, Iyed Salhi, Jihen Khalfoun, Mohaned Omar, Cain C. T. Clark, Bogdan-Constantin Ungurean, Urs Granacher, Hassane Zouhal

**Affiliations:** 1 Higher Institute of Sport and Physical Education of Ksar-Said, University of Manouba, Tunis 1000, Tunisia; abderraouf.benabderrahman@issep.uma.tn (A.B.A.); jihenkh9@gmail.com (J.K.); mohanad_omar8@hotmail.com (M.O.); 2 Tunisian Research Laboratory “Sports Performance Optimization” LR09SEP01, National Center of Medicine and Science in Sports (CNMSS), Tunis 1000, Tunisia; alouiali1991@gmail.com; 3 Research Unit: “Sport Sciences, Health and Movement”, High Institute of Sport and Physical Education of Kef, University of Jendouba, Kef 7100, Tunisia; jnidhal@gmail.com (N.J.); iyedsalhi38@gmail.com (I.S.); 4 College of Life Sciences, Birmingham City University, Birmingham B15 3TN, UK; cainctclark@gmail.com; 5 Faculty of Physical Education and Sports, Alexandru Ioan Cuza University, 700552 Iasi, Romania; bungurean@yahoo.com; 6 Exercise and Human Movement Science, University of Freiburg, 79102 Freiburg, Germany; 7 M2S (Movement Sport Science Laboratory), University of Rennes, 35044 Rennes, France; 8 Institut International des Sciences du Sport (2I2S), 35850 Irodouer, France

**Keywords:** intermittent exercise, anthropometric variation, repeated sprint sequences, vertical-jump tests

## Abstract

Objective: We examined the effects of eight weeks of single-mode resistance, repeated sprint training, and the combination of the two programs on selected measures of physical fitness (muscle power, speed, and body composition), hematological parameters, and plasma volume variations in highly trained soccer players. Sixty male soccer players from the Tunisian national Ligue were randomly allocated to a resistance training group (RTG), a repeated sprint training group (RSTG), a combined resistance and repeated sprint training group (CTG), or an active control group (CG, soccer training only). The training volumes were similar between groups. Before and after training, we examined body composition, squat jump (SJ), countermovement jump (CMJ), sprint 30 m (S30), repeated-sprint sequences (RSSs), hematocrit, hemoglobin, mean hemoglobin concentration (MHC), and plasma volume. Significant group-by-time interactions were recorded for the RSS indices, SJ, and S30 (*p* < 0.039], 0.1< ηp^2^ < 0.49]), as well as the hematological parameters (*p* = 0.0001–0.045, 0.11 < ηp^2^ < 0.46). In terms of physical fitness, using post hoc tests, the CTG showed significantly greater gains compared to the RSTG, RTG, and the CG on the best time index of the RSSs (*p* = 0.008; d = 4.1), SJ (*p* = 0.004; d = 4.18) and 30 m linear sprint time (*p* = 0.008; d = 3.84). Body fat percentage also decreased significantly in the CTG compared to all other groups (*p* < 0.005, 0.21 < d< 0.35). Regarding hematological parameters (i.e., hemoglobin and hematocrit), the CTG, RSTG, and RTG showed significant decreases (*p* < 0.05) in their hemoglobin and hematocrit values compared to the CG (*p* < 0.05, 0.11 < d< 2.22]). Eight weeks of combined training compared to single-mode training was found to be more effective in improving fitness measures in highly trained soccer players. However, there appeared to be no consensus regarding the effect of single and combined repeated-sprint and resistance training on the hematological system.

## 1. Introduction

There is evidence that different training protocols have the potential to improve athletes’ physical fitness [1]. For instance, repeated-sprint ability (RST) and resistance training (RT) appear to be particularly effective in improving soccer players’ physical fitness [2]. Of note, repeated-sprint ability is considered to be the capability to conduct successive sprints with minimal recovery in between, over a series of sprints, with the objective of enhancing speed and maximal oxygen uptake [3]. RT involves exercises with different resistive loads, movement velocities, and training modalities, primarily aimed at enhancing measures of muscle strength and power [4].

Previously, many research groups have examined the effectiveness of different resistance training types, particularly plyometric jump training on young soccer players’ physical fitness [3,5].

Researchers who have previously examined the effects of RT on soccer players’ physical fitness have primarily employed repetitions to failure (XRM) or high intensities (70–95% 1 RM), often with athletes with no prior RT experience [6,7]. Furthermore, heavy resistance strength training (3–6 RM) is rarely performed during the competitive soccer season, because it induces excessive fatigue that may compromise the effectiveness of soccer-specific technical and tactical training following this high-intensity exercise type [8].

Both RST and RT are widely applied exercise regimes in soccer because they have been proven to enhance physical fitness and physiologic adaptations, such as maximal oxygen uptake, lactate transport capacity, and phosphocreatine recovery [9,10,11,12]. Yet, little information exists with regard to the combined effects of RST and RT on physical fitness and physiological responses, including hematological changes [13,14].

Training-induced adaptations following RT do not only induce improvements in measures of physical fitness and sport-specific performance, but also in the hematological system. For instance, there is evidence that RT has an impact on hemoglobin, hematocrit, and plasma volume [15]. Training-induced hemoglobin changes may improve O_2_ transportation, resulting in more efficient muscle oxygenation [15]. Training-related improvements in hematocrit levels increase the amount of red blood cells, which again has a positive impact on oxygen transport capacity [16]. Training-induced changes in the plasma volume primarily set in if the training intensity is high, which is associated with a relative dilution of plasma, causing deterioration in the muscle hematocrit and hemoglobin concentrations [14]. The regulation of these three hematological parameters is crucial to maintain peak performance and prevent the negative signs of overtraining [17].

According to findings from a systematic review by Saidi et al. [17], changes in hematological parameters were unrelated to training-related performance changes in professional male soccer players aged >17 years. Moreover, based on data from original research, Saidi et al. [18] could not find significant correlations between changes in hematological markers and changes in physical performance in professional male soccer players after a six-week in-season period interspersed with 10 soccer matches. Furthermore, Silva et al. [19] were unable to find significant correlations between performance at the anaerobic threshold and hematological parameters (e.g., erythrocytes concentration, hemoglobin, and hematocrit) in Brazilian soccer players. Yet, the same researchers showed that long-term soccer training induced greater alterations in hematological markers. Due to discrepancies in such findings and the few available studies, Saidi et al. [17] recommended interpreting findings on a potential relation between physical performance and hematological parameters with caution. Therefore, it is timely to explore the effects of different training protocols on measures of physical fitness and hematological responses in soccer players.

Considering the limited scientific knowledge on the effects of combined RT and RST versus single-mode RT or RST on soccer players’ physical fitness and physiological parameters, the purpose of this study was to investigate the effects of an eight-week combined RT and RST program versus single-mode RT or RST, alongside an active control group, on the physical fitness and physiological responses of professional soccer players. Selected measures of physical fitness were assessed using the squat and countermovement jump tests, as well as sprint tests, while physiological responses included hematocrit, hemoglobin, mean hemoglobin concentration, and plasma volume. It was hypothesized that the combined RT and RST program would be more effective in changing the players’ physical fitness and hematological parameters compared to the single-mode training modalities and soccer training only (active control) [11,12].

## 2. Materials and Methods

### 2.1. Participants

**Figure 1:**
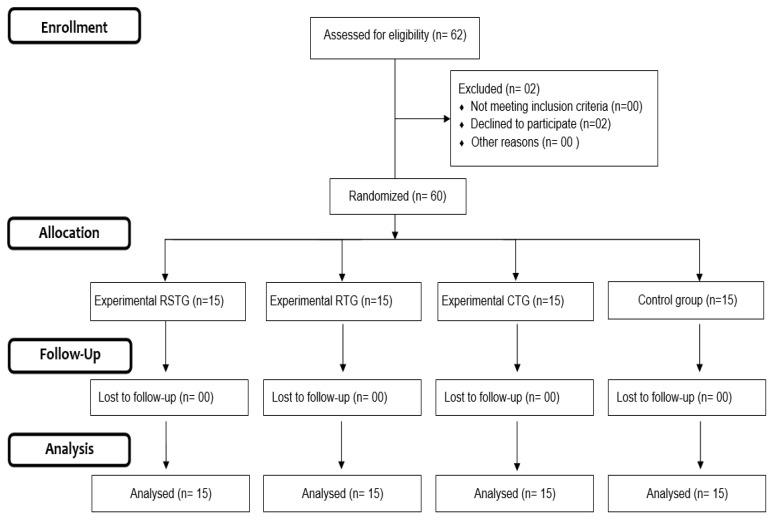
Consort flow diagram.

During the preparatory period of the soccer season, 62 highly trained male soccer players [13] (30 adult soccer players and 32 under twenty-one players [U21]) from the same Tunisian soccer team (Kef Olympic) were enrolled in this study. The highly trained and U21 teams competed in the 3rd Tunisian soccer league. Two players were excluded from the final data analysis because their training attendance was below 85% of the scheduled training sessions (Figure 1). Accordingly, full data sets were obtained from 60 players aged 21.8 ± 2.6 years (body mass: 67.5 ± 7.4 kg; height: 179.6 ± 7.8 cm; BMI: 21.5± 2.5 kg/m^2^). Players were randomly allocated to the following four groups: (a) a resistance training group (RTG; *n* = 15), (b): a repeated-sprint training group (RSTG: *n* = 15); (c) a combined repeated-sprint training and resistance training group (CTG; *n* = 15); and (d) an active control group (CG; *n* = 15). The participants were randomly assigned to these four groups using a free online tool (https://www.randomizer.org; 23 February 2021).

There were no significant between-group baseline differences for anthropometric data (i.e., body height, leg length, body mass, and BMI). None of the participants reported any recent (three months prior to the start of the study) history of hip, knee, or ankle injuries. The players trained ten sessions per week for approximately two hours per session (overall 20 h of training per week) during the preparatory part of the season. Prior to participation, all players provided their written informed consent outlining the commitment, benefits, potential risks, and procedures of the study. The study was conducted in accordance with the latest version of the Declaration of Helsinki and was approved by the local Ethics Committee of the University of Manouba, Tunis, Tunisia, (No. 0375/2021, on 16 February 2021).

### 2.2. Procedures

The study was carried out over an eight-week intervention period and was designed to examine the effects of three different training programs on measures of physical fitness, including body composition, plasma volume variations, and hematological parameters. Players from the different experimental groups were evaluated twice during the study; the first test session (T1) took place in the beginning of October, while the second test session (T2) was performed at the end of November. Measures of physical fitness, plasma volume, and hematological parameters were evaluated over three days. On the first day, blood samples were drawn to determine the resting plasma volume and hematological parameters. On the second day, the participants performed two physical fitness tests, the squat jump (SJ) and the countermovement jump (CMJ). On the third test day, the participants performed a 30 m linear sprint test and repeated-sprint ability test. The physical fitness tests applied were familiar to all players, because they are regularly conducted during the soccer season. All anthropometric and physical fitness tests took place in the morning (11 ± 1 h), 3 h after a standardized breakfast (10 kcal/kg; 55% of carbohydrates; 33% from lipids; 12% from proteins) determined by an experienced nutritionist. The players were asked to standardize and follow the same nutritional and hydration guidance before each test session to minimize dietary impacts on performance changes.

### 2.3. Training Programs

The usual training was performed from Tuesday to Saturday and included, on average, 20 h of training per week during the preparatory part of the season, with each session lasting 90 min (Table 1). Partial recovery (i.e., full day of morning or afternoon) was provided on Tuesday, Saturday, Thursday, and Sunday. Alongside their usual training, the players participated in specific exercise programs three times a week (Monday, Wednesday, and Friday) on non-consecutive days (48 h rest) for eight weeks, using repeated sprints training only (for RSTG), back squat training only (RTG), or combined repeated-sprint and resistance training (CTG). The descriptive characteristics of the training programs are presented in Table 2. The training sessions lasted 15–20 min for RSTG, RTG, and CTG and were conducted before the field training. The warm-up program, for all sessions, included 10 min of submaximal running with exercises of vertical and horizontal jumps, dynamic stretching, and sprint-specific drills. All training sessions were supervised by researchers, physiotherapists, or coaches. The active control group performed their regular and soccer-specific training over the 8-week intervention period (Table 2). The intervention groups replaced soccer-specific exercises with their specific intervention programs. The training volumes were similar across the four groups.

The players reported their rate of perceived exertion approximately thirty minutes after each training session. The rate of perceived exertion was assessed using Borg’s 6–20 scale of perceived exertion (i.e., very, very light to very, very hard) [20].

The training load (Figure 1) was calculated, per week, using the following formula:

Training Load = Dmean × RPEmean

Dmean represents the session mean duration (min) per week and RPEmean represents the mean rate of perceived exertion per week.

### 2.4. Anthropometric Measurements

The participants’ body mass was measured to the nearest 0.1 kg using an electronic scale (Kern, MFB 150K100, Balingen, Germany). During testing, the participants were lightly dressed and weighed barefoot on the scale. Their body height was measured to the nearest 0.5 cm using a tape measure fixed to the wall. Their body mass index (BMI) was calculated by dividing their body mass (kg) by the square of their height (m) (kg/m^2^). Lean body mass and body fat percentage were determined using a multifrequency bioelectrical impedance device (Inbody 720, Seoul, Republic of Korea). During testing, the athletes were kindly asked to stand upright and to grasp the handles of the analyzer, thereby providing contact with a total of eight electrodes, which allowed for a segmental analysis of the trunk, left and right arms, and left and right legs. All measurements were performed by an independent investigator in the morning in a fasted state, according to protocols described by the International Biological Program [21].

### 2.5. Physical Fitness Tests

#### 2.5.1. Muscle Power

During SJ, the players jumped from a semi-squatting position (90° knee flexion) without countermovement. During CMJ, the players started in a standing position and performed an explosive vertical jump using a slow stretch-shortening cycle (SSC) at ~90° knee flexion. For both tests, the players were instructed to jump as high as possible. All players performed their vertical jumps with the hands kept on the hips throughout the test. Each test was measured with three trials, with a 60 s interval between each trial. The average of the best three trials was taken for further analysis. Vertical jump height was evaluated using an opto-electric system (Opto-Jump, Microgate, Italy). Pilot data from 60 players collected on two different days were used to determine the reproducibility of the SJ and CMJ tests (SJ: ICC = 0.848; CMJ: ICC = 0.902).

#### 2.5.2. Speed

All groups performed a standard 10 min jog warm-up. This was followed by two sprints over 20 m, recorded by paired photocells (Globus, Microgate, Bolsano, Italy). Three trials were separated by 6–8 min of recovery for each sprint test. The players began from a standing position just before the starting photocell beam. The best trial was taken for further analysis. Pilot data from 60 players collected on two different days were used to determine the reproducibility of the test (ICC = 0.830).

The repeated-sprint sequences (RSSs) test consisted of two sequences of 5 × 20 m maximum sprints, with 15 s of active rest between the sprints and a recovery period of one min between the sequences [22]. The RSS performance indices were the total time (TT), best time (BT), and fatigue index (FI), as proposed by Fitzsimons [23]: [TT/(Tpic × number of sprint) × 100) − 100)]. The RSS performances were evaluated using an electronic timing system (Globus, Microgate, Bolsano, Italy). Pilot data from 60 players collected on two different days were used to determine the reproducibility of the test for each index (ICC = 0.806–0.996).

#### 2.5.3. Blood Analyses

Before and after the intervention period, blood samples were taken at rest in the morning (11.00 am ±1 h), after 12 h of overnight fasting and 24 h from the last bout of exercise. For each player, 0.5 mL of a venous blood sample was collected before and after the eight-week training program. Blood collection was performed through an antecubital vein via a heparinized catheter (Insyte-W ^TM^, 1.1 mm diameter × 30 mm, Becton Dickinson, Egypt) in the laboratory. The samples were immediately cooled in an ice bath and then centrifuged. The obtained plasma aliquots were stored at −80 °C until further analysis.

Hematocrit (Ht), hemoglobin (Hb), and mean hemoglobin concentration (MHC) were automatically determined at the same time and from the same blood sample by using hemoglobin standard laboratory procedures. The hematological parameters were generally analyzed within 3 h using a multichannel automated hematology analyzer (Sysmex XS-1000i, CompuGroup Medical, Mesa, AZ, USA).

Plasma volume variation (PVV) was estimated according to Ht and Hb data using the following formula [24]:

PVV (%) = 100 × (Hb_2_/Hb_1_ × ((1 − Ht_1_))/((1 − Ht_2_))) – 100

Ht_1_ = hematocrit pre training

Ht_2_ = hematocrit post training

Hb_1_ = hemoglobin pre training

Hb_2_ = hemoglobin post training.

## 3. Statistical Analyses

Data are expressed as means and standard deviations (SDs). The normality of the data was assessed and confirmed using the Kolmogorov–Smirnov test.

A two-way analysis of variance for repeated measures (ANOVA) was conducted, considering four groups (CG, RSTG, RTG, and CTG) and two time points (pre and post) for each parameter and test (SJ, CMJ, S30, RSS, hematocrit, hemoglobin, and MHC). Bonferroni-adjusted post hoc tests were computed.

Partial eta squared (ηp^2^) values were extracted from the ANOVA output, and Cohen’s d effect sizes (d) were calculated to quantify meaningful differences in the data following Cohen’s guidelines (1988), which classify effects as trivial (<0.2), small (0.2–0.59), medium (0.60–1.19), large (1.2–1.99), or very large (≥2.0) [25]. Performance changes (∆%) between conditions were calculated according to the following equation: ((condition values 2 − condition values 1)/condition values 1) × 100. Statistical significance for all analyses was set, a priori, at *p* < 0.05. Data were analyzed using the SPSS 28 software package (SPSS Inc., Chicago, IL, USA).

## 4. Results

All soccer players received treatments as allocated. At pre-program testing, no between-group differences were found for any of the variables analyzed.

### 4.1. Physical Fitness

Training-induced changes in physical fitness are presented in Table 3.

For the TT index, a significant group-by-time interaction (*p* < 0.0001; ηp^2^ = 0.41), main effect of time (*p* < 0.0001; ηp^2^ = 0.81), and main effect of group (*p* = 0.022; ηp^2^ = 0.08) were recorded. Bonferroni-adjusted post hoc tests revealed significant (*p* < 0.001; 0.53 < d < 1.29) pre-to-post reductions for the TT index in all groups.

Contrast analysis revealed a significant decrease for the CTG vs. control group (*p* = 0.037; d = 3.05), RSTG vs. control group (*p* = 0.015; d = 3.56), RSTG vs. RTG (*p* = 0.021; d = 3.39), and RTG vs. CTG (*p* = 0.049; d = 2.88). However, no significant changes were observed between the control group vs. RTG or between the RSTG vs. CTG (*p* > 0.05).

For the BT index, a significant group-by-time interaction (*p* = 0.07; ηp^2^ = 0.1) and main effect of time (*p* = 0.006; ηp^2^ = 0.07) were recorded. However, no significant main effect of group (*p* = 0.055; ηp^2^ = 0.06) was found. Bonferroni-adjusted post hoc tests revealed significant (*p* < 0.001; 0.29 < d < 0.61) pre-to-post reductions for the BT index in all groups.

Contrast analysis revealed a significant decrease for the CTG vs. control group (*p* = 0.008; d = 4.1) and CTG vs. RSTG (*p* = 0.046; d = 3.3). However, no significant change was observed between the control, RT, and RST groups.

For the FI index, a significant group-by-time interaction (*p* < 0.0001; ηp^2^ = 0.49) and main effects of time (*p* = 0.0001; ηp^2^ = 0.41) were recorded. However, no significant main effect of group (*p* = 0.959; ηp^2^ = 0.003) was found. Bonferroni-adjusted post hoc tests revealed significant (*p* < 0.001; 0.03 < d < 0.07) pre-to-post reductions for the FI index in all groups.

Contrast analysis revealed a significant decrease in FI after the training program (*p* < 0.0001; 0.4 < d < 0.9).

Regarding SJ performance, the analysis revealed a significant group-by-time interaction (*p* = 0.039; ηp^2^ = 0.11), main effect of time (*p* = 0.003; ηp^2^ = 0.11), and main effect of group (*p* = 0.026; ηp^2^ = 0.123). Bonferroni-adjusted post hoc tests showed significant (*p* < 0.001; 0.79 < d < 1.01]) pre-to-post improvements in SJ performance in all groups.

Contrast analysis revealed a significant increase in vertical height in the CTG vs. control group (*p* = 0.004; d = 4.18).

Regarding CMJ performance, no significant group-by-time interaction (*p* = 0.232; ηp^2^ = 0.06) or main effect of group (*p* = 0.026; ηp^2^ = 0.123) were found. However, significant main effects of time were observed (*p* < 0.0001; ηp^2^ = 0.304).

Regarding S30 performance, a significant group-by-time interaction (*p* = 0.001; ηp^2^ = 0.19), main effect of time (*p* < 0.0001; ηp^2^ = 0.43), and main effect of group (*p* = 0.039; ηp^2^ = 0.11) were found. Bonferroni-adjusted post hoc tests revealed significant (*p* < 0.004; d = 0.17 < d < 0.37) pre-to-post improvements in S30 performance in all groups.

Contrast analysis indicated a significant decrease in sprint time in the CTG compared to the control (*p* = 0.008; d = 3.84) and RTG (*p* = 0.039; d = 2.97).

### 4.2. Anthropometric Variation

The mean (±SD) data for body composition measured before and after the different training programs for the four groups are presented in Table 4. A significant decrease in body fat (%) was observed in the RSTG (*p* = 0.05; d = 0.22) and CTG (*p* = 0.05; d = 0.23) compared to the other programs.

### 4.3. Hematological Parameters

The hematological parameters measured before and after training are represented in Table 5.

For hemoglobin, a significant group-by-time interaction (*p* < 0.0001; ηp^2^ = 0.23), main effect of time (*p* < 0.0001; ηp^2^ = 0.33), and main effect of group (*p* < 0.0001; ηp^2^ = 0.6) were found. Bonferroni-adjusted post hoc tests revealed a significant pre-to-post decrease in hemoglobin in the RSTG (*p* < 0.001; d = 0.19) and control (*p* < 0.001; d = 0.17).

Contrast analysis showed a significant increase in hemoglobin for the control group compared to the CTG (*p* < 0.0001; d = 7.55), RTG (*p* < 0.0001; d = 2.22), and RSTG (*p* < 0.0001; d = 4.22).

For hematocrit, a significant group-by-time interaction (*p* = 0.045; ηp^2^ = 0.11), main effect of time (*p* < 0.0001; ηp^2^ = 0.70), and main effect of group (*p* < 0.0001; ηp^2^ = 0.29) were found. Bonferroni-adjusted post hoc tests revealed a significant (*p* < 0.001–0.004) pre-to-post decrease in hematocrit in the RTG (*p* < 0.001; d = 1.45) and CTG (*p* < 0.001; d = 2.93).

Contrast analysis revealed a significant increase in hematocrit with RSTG compared to the control (*p* < 0.0001; d = 2.41), RTG (*p* < 0.0001; d = 2.22), and CTG (*p* < 0.0001; d = 2.22).

For MHC, a significant group-by-time interaction (*p* < 0.0001; ηp^2^ = 0.46), main effect of time (*p* < 0.0001; ηp^2^ = 0.76), and main effect of group (*p* = 0.001; ηp^2^ = 0.21) were found. Bonferroni-adjusted post hoc tests revealed a significant (*p* < 0.004) pre-to-post decrease in hematocrit in the RSTG (*p* < 0.001; d = 0.38), control group (*p* < 0.001; d = 0.95), and RTG (*p* = 0.012; d = 0.29).

Contrast analysis indicated a significant increase in hematocrit in the RSTG compared the RTG (*p* = 0.028; d = 2.21) and CTG (*p* < 0.0001; d = 2.46). Also, there was a significant decrease in the CTG compared to control group(*p* < 0.001; d = 2.01)

For plasma volume variation, using one-way ANOVA, a significant difference was observed between groups (*p* = 0.013; d = 0.83). Bonferroni-adjusted post hoc tests revealed a significant increase in plasma volume in the RSTG compared to the control group (*p* = 0.022; d = 0.49) and RTG (*p* = 0.002; d = 0.82).

## 5. Discussion

The main purpose of this study was to investigate the effects of an eight-week combined training program versus single-mode resistance and sprint training programs on the body composition, physical fitness, and hematological parameters in highly trained male soccer players. Concordant with our research hypothesis, the results of the present study indicate that the combined repeated-sprint and resistance training program had a significant impact on the anthropometric indices, physical fitness, hematocrit (%), and MHC (%) of the soccer players.

In contrast, the RT alone led to improvements only in SJ, CMJ, and the 30 m sprint, with no changes observed in hematological parameters or anthropometric measurements. Additionally, the repeated-sprint training program improved the performance only in the total time index of RSS, with no changes in body fat composition.

Regarding body composition, the soccer players undergoing combined training demonstrated beneficial changes in their anthropometric indices compared to the RTG and active control group. In fact, the results of the present study suggest that combined training programs, including resistance and repeated-sprint exercises, may be more effective than resistance training alone in reducing anthropometric measurements, especially for body fat percentage.

The results of this study showed that CTG significantly improved the 30 m sprint, TT, and BT indices of RSS, while RST improved only the S30 and BT indices of RSS. However, no significant change was observed in the RSS indices and S30 with RT. In accordance with our current findings, previous studies have reported that CTG elicited significant improvements in repeated-sprint performance indices in futsal and soccer players [26,27], although some studies reported that CTG had no effect on repeated sprint performances. Therefore, the results from the combined training group of the current study correspond with earlier research [26,27], in that both resistance and repeated-sprint training for 6–8 weeks can result in an enhancement in the ability to perform repeated sprints with brief recovery times. This finding can be explained by the specific characteristics of soccer training that require both RT and RST to improve the skills of strength and speed during repeated sprinting bouts [26].

Soccer players undergoing CTG in the present study demonstrated an increase in SJ and CMJ compared to the control group. A comparable significant improvement was observed in the RTG on the SJ and CMJ tests. Our results are similar to previous studies that reported that combined high-intensity strength and speed training [26] or resistance training [27] resulted in an increased vertical jump (SJ and CMJ) performance, although some studies have reported that resistance training alone [10,28] is not sufficient to elicit an increase in SJ and CMJ performances. These disparities in results can be explained by variations in certain factors, such as the specificity of training programs [29], learning effects [28], the number of matches per week, and the frequency of weekly training sessions [30]. Therefore, all vertical jumps could be expected to improve with RT. However, the SJ and CMJ performances improved to a greater extent in the CTG than in the RTG (SJ: 3.9% vs. 2.8%; CMJ: 4.5% vs. 4%). In addition, it is well documented that the combination of resistance training and repeated-sprint training could impact vertical jumps (SJ and CMJ) in a comparable manner to combined resistance and plyometric training [31].

The current results showed a significant decrease in hematological parameters after training programs. In agreement with our results, previous studies have suggested that Hb and Ht decrease after training or competitive periods [18,32]. Notably, Saidi, et al. [18] reported significant decreases in Hb and Ht after 6 weeks of congested match play in elite soccer players. In contrast, in the same study, Saidi, et al. [18] also observed a significant decrease in PVV, with no change in MHC. However, other findings suggest that competitive periods with a variety of training loads maintain hematological values [33]. These differences in findings can primarily be explained by psychological factors, training intensity, match frequency, duration, environmental conditions, and players’ expertise level.

Within experimental groups, to our knowledge, no studies have examined the effects of different training programs on hematological parameters in soccer players yet. However, Cavalcante da Silva, et al. [19] showed that Ht could be affected by training volume over a 6-week soccer training program. This evidence is consistent with the reactivity of hematological parameters to variations in training volume observed in the present study.

Our results showed that eight weeks of training resulted in significant Hb level decreases in the RSTG, Ht level reductions in the RTG and CTG, and a PVV decrease increase in the RSTG. To our knowledge, no study has investigated the specific or combined effects of RSTG and RTG training on the hematological parameters in U21 soccer players. In fact, these results deserve to be further investigated in order to explain the causes of such reductions, despite the significant improvements observed for physical performance in the RSS test.

In fact, Anđelković et al. [34] reported that, during the competitive phase, sports anemia was observed after 90 days of soccer training in elite players, represented by a significant increase of 7.5% in PVV and a decrease in Hb and Ht levels. These last authors observed that the idea of sports anemia supports a decrease in Ht and Hb values during competition periods [34]. Theoretically, sports anemia is considered to be the first sign of regular overtraining and can, in some cases, be responsible for a sharp decline in endurance performance [35], which was not the case in our study. In divergence from the present study, as reported by Silva et al. [19], increases in hemoglobin and hematocrit are related to improvements in physical performance. The summary of research on changes in hematological parameters and their relationships with physical performance should be considered with caution. Indeed, such methodological inconsistencies between studies regarding the duration of training periods, workload (intensity and volume), the specific characteristics of training programs, and the time of investigation (preparatory or competition phase) explain the contradictory results present in these studies.

## 6. Limitations

Our study has some limitations, as follows: (1) only a limited number of elite male soccer players were included in our study, making it challenging to generalize our results or to extrapolate them to female soccer players; (2) an indirect method was used to calculate PVV, and the use of a direct method (e.g., blue Evans) would be more accurate; and (3) the hydration status of the players was only standardized during the training sessions and on the days of testing and blood sampling, not during the entire period of the experiment (e.g., outside of training times), which may have had an impact on the results. However, the players were advised to maintain normal hydration.

## 7. Conclusions

In conclusion, the current results demonstrate that eight weeks of combined resistance and repeated-sprint training elicited a significant improvement in vertical jump, sprint, and repeated sprint sequences to a greater extent than resistance or repeated-sprint training alone. Moreover, these changes were related to some hematological parameters, plasma volume, and changes in body composition.

Regarding practical application, this study can provide coaches and fitness trainers with useful information for using combined resistance/repeated-sprint training in their practice in order to take full advantage of the favorable transfer during the preparatory phase. The application of this combined mode as a best practice is suggested for achieving the optimal anaerobic performance during this preparatory phase. However, hematological parameters should be carefully considered and controlled due to possible high-degree deteriorations that could have adverse effects in the competitive phase—which is prolonged by an increase in the number of matches.

## Figures and Tables

**Table 1:** Example of a weekly soccer training program.

	Monday	Tuesday	Wednesday	Thursday	Friday	Saturday	Sunday
**Morning** **session**	Technical training	Rest recovery	Technical training	Rest recovery	Technical training	Technical training	Rest recovery
**Afternoon session**	Normal or specific physical conditioning *	Technical training	Normal or specific physical conditioning *	Technical training	Normal or specific physical conditioning *	Rest recovery	Friendly match

*: repeated sprints training only or back -squat training only or combined repeated- sprint and resistance training.

**Table 2:** The physical conditioning programs of the three experimental groups.

8 Weeks of Training Programs								
	1	2	3	4	5	6	7	8
RSTG	2 series (5 × 20 m); r = 15 s; R = 1 min	2 series (5 × 20 m); r = 15 s; R = 1 min	10 × 20 m; r = 15 s	10 × 20 m; r = 15 s	2 series (5 × 20 m shuttle); r = 15 s; R = 1 min	2 series (5 × 20 m shuttle); r = 15 s; R = 1 min	10 × 20 m shuttle; r = 15 s; R = 1 min	10 × 20 m shuttle; r = 15 s
RTG	3 × 15 repetitions 60–65% 1-RM; R = 1 min	3 × 15 repetitions 60–65% 1-RM; R = 1 min	3 × 15 repetitions 65–70% 1-RM; R = 1 min	3 × 15 repetitions 65–70% 1-RM; R = 1 min	3 × 15 repetitions 70–75% 1-RM; R = 1 min	3 × 15 repetitions 70–75% 1-RM; R = 1 min	3 × 15 repetitions 75–80% 1-RM; R = 1 min	3 × 15 repetitions 70–75% 1-RM; R = 1 min
CTG	repeated sprint: 2 series (5 × 20 m); r = 15 s; R = 1 min	back-squat: 3 × 15 repetitions 60–65% 1-RM; R = 1 min	repeated sprint: 10 × 20 m; r = 15 s	back-squat: 3 × 12 repetitions 65–70% 1-RM; R = 1 min	repeated sprint: 2 series (5 × 20 m S); r = 15 s; R = 1 min	back-squat: 3 × 10 repetitions 70–75% 1-RM; R = 1 min	repeated sprint: 10 × 20 m shuttle; r = 15 s; R = 1 min	back-squat: 3 × 8 repetitions 70–75% 1-RM; R = 1 min

RSTG: repeated-sprint training group; 1-RM: one repetition maximum; RTG: resistance training group; CTG: combined training group; r: passive recovery; S: shuttle and R: rest.

**Table 3:** Changes in performance in physical fitness tests after the 8 weeks of training.

	RSS TT (s)		RSS BT (s)		RSS FI (%)		SJ (cm)		CMJ (cm)		Sprint 30 m (s)	
	Pre	Post	Pre	Post	Pre	Post	Pre	Post	Pre	Post	Pre	Post
Control	33.70 ± 0.64	33.39 ± 0.53	3.15 ± 0.07	3.13 ± 0.07	6.82 ± 2.2	6.76 ± 2.1	28.9 ± 2.38	29.65 ± 2.9	38.7 ± 3.1	39.38 ± 3.1	4.59 ± 0.22	4.55 ± 0.26
RSTG	33.60 ± 0.64	32.67 ± 0.6 ¥	3.12 ± 0.08	3.11 ± 0.06	6.62 ± 2.1	6.72 ± 2.11	29.83 ± 2.2	29.1 ± 1.9	37.21 ± 1.8	39.03 ± 2.7	4.62 ± 0.19	4.55 ± 0.19 ¥
RTG	33.79 ± 0.50	33.05 ± 0.64 φ	3.14 ± 0.07	3.1 ± 0.09	6.8 ± 2	6.72 ± 2.11	30.3 ± 1.1	31.2 ± 2.1 £	39.34 ± 3.6	40.86 ± 1.1	4.56 ± 0.2	4.41 ± 0.2 £
CTG	33.67 ± 0.61	32.02 ± 0.73 ‡θ	3.13 ± 0.06	3.04 ± 0.2	6.8 ± 1.5	6.67 ± 2.12	30.81 ± 1.29	32.07 ± 1.72 ‡	39.32 ± 1.3	41.08 ± 2.08	4.49 ± 0.2	4.33 ± 0.21 ‡μ
Time effect	0.0001 (0.81) ***		0.006 (0.07) **		0.0001 (0.41) ***		0.003 (0.113) **		0.0001 (0.304) ***		0.0001 (0.43) ***	
Group effect	0.022 (0.082)		0.055 (0.065)		0.959 (0.003)		0.026 (0.12) *		0.140		0.039 (0.11) *	
Group × time interaction	0.0001 (0.41) ***		0.007 (0.1) **		0.0001 (0.49) ***		0.039 (0.109) *		0.232 (0.058)		0.001 (0.193) **	

RSTG: repeated-sprint training group; RTG: resistance training group; CTG: combined training group., RSS: repeated-sprint sequences, SJ: squat jump, CMJ: countermovement jump, TT: total time, BT: best time, and FI: fatigue index. Interaction group × time: *p* < 0.01; Contrast analysis: ¥ Control vs. RSTG, ‡ CTG vs. control, μ CTG vs. RSTG, £ RTG vs. control, φ RTG vs. RSTG, and θ RTG vs. CTG. *: *p* < 0.05, **: *p* < 0.01, and ***: *p* < 0.001.

**Table 4:** Effects of different training programs on soccer players’ body mass index (BMI), body fat percentage (BFP), and lean body mass (LBM).

		Control	RSTG	RTG	CTG
Body mass (kg)	Pre	73 ± 4.3	75.6 ± 5.1	72.3± 4.8	72.6± 5.1
	Post	71.8 ± 5.1	73.2 ± 5.1	72.1 ± 5	70.3 ± 5.1 ‡μ€*
BMI (kg/m^2^)	Pre	22.8 ± 1.2	24.4 ± 1.28	22.7 ± 1.2	22.9 ± 1.2
	Post	22.6 ± 1.1	23.2 ± 1.3	22.6 ± 1.1	21.4 ± 1.4 ‡μ€*
BF (%)	Pre	15 ± 1.3	14.9 ± 2.0	15.1 ± 1.5	14.7 ± 2.9
	Post	14.9 ± 1.8	14.7 ± 2.3 ¥∞	15.0 ± 1.8	14.2 ± 2.4 ‡μ€*
LBM (kg)	Pre	66.5 ±4.3	65.6 ±4.3	66.3 ±4.3	66.6 ±4.3
	Post	65.9± 4.7	66± 4.7	65.8 ± 4.6	65.4± 4.7

RSTG: repeated sprint training group; RTG: resistance training group; CTG: combined training group; r: passive recovery; BMI: Body mass index, BF: body fat, and LBM: lean body mass. *: *p* < 0.05; Contrast analysis: ‡ CTG vs. control, μ CTG vs. RSTG, € RTG vs. CTG, ¥ Control vs. RSTG, and ∞ RTG vs. RSTG.

**Table 5:** Effects of three exercise types and active control on soccer players’ hematological parameters.

	Hemoglobin (g/dL)		Hematocrit (%)		MHC (%)		PVV (T1-T2) (%)
	Pre	Post	Pre	Post	Pre	Post	
Control	14.85 ± 0.1	14.8 ± 0.4	43.9 ± 1	42.6 ± 1.2	34.5 ± 2.2	32.7 ± 1.5	1.23 ± 1
RSTG	14.4 ± 0.2	14.3 ± 0.7 ¥	45.5 ± 0.4	42.7 ± 2.7 ¥	33.9 ± 1.1	33.5 ± 1 φ	3.54 ± 2.56 ¥φ
RTG	14.2 ± 0.5	14 ± 0.2 £	43.9 ± 1.2	42.8 ± 0.9 £	33.4 ± 3.8	32.5 ± 2.2	−0.30 ± 2.85
CTG	14.7 ± 0.6	14.5 ± 0.5 ‡€	45.7 ± 0.5	43 ± 1.2 €	34.1 ± 1.6	31.4 ± 1.4 ‡€	1.62 ± 4.60
Time effect	0.0001 (0.33) ***		0.0001 (0.70) ***		0.0001 (0.76) ***		---
Group effect	0.0001 (0.6) ***		0.0001 (0.29) ***		0.001 (021) **		0.0001(0.83) ***
Group × time interaction	0.0001 (0.23) ***		0.045 (0.11) *		0.0001 (0.46) ***		----

MHC: Mean hemoglobin concentration; PVV: plasma volume variation; RSTG: repeated-sprint training group; RTG: resistance training group; and CTG: combined training group. Interaction group × time: *: *p* < 0.05, **: *p* < 0.01, and ***: *p* < 0.001; Contrast analysis: ¥ Control vs. RSTG; ‡ CT vs. control; £ RTG vs. control; € CT vs. RSTG; and φ RTG vs. RSTG.

## Data Availability

The raw data supporting the conclusions of this article will be made available by the authors on request.

## References

[1] Xiao W., Soh K.G., Wazir M.R.W.N., Talib O., Bai X., Bu T., Gardasevic J. (2021). Effect of functional training on physical fitness among athletes: A systematic review. Front. Physiol..

[2] Stølen T., Chamari K., Castagna C., Wisløff U. (2005). Physiology of Soccer. Sports Med..

[3] Meylan C.M.P., Cronin J.B., Oliver J.L., Hughes M.G., Manson S. (2014). An Evidence-Based Model of Power Development in Youth Soccer. Int. J. Sports Sci. Coach..

[4] Rampinini E., Coutts A.J., Castagna C., Sassi R., Impellizzeri F.M. (2007). Variation in Top Level Soccer Match Performance. Int. J. Sports Med..

[5] Ramirez-Campillo R., García-Hermoso A., Moran J., Chaabene H., Negra Y., Scanlan A.T. (2022). The effects of plyometric jump training on physical fitness attributes in basketball players: A meta-analysis. J. Sport Health Sci..

[6] Tomljanović M., Spasić M., Gabrilo G., Uljević O., Foretić N. (2011). Effects of five weeks of functional vs. traditional resistance training on anthropometric and motor performance variables. Kinesiology.

[7] Wong Y.M., Ng G. (2010). Resistance training alters the sensorimotor control of vasti muscles. J. Electromyogr. Kinesiol..

[8] Draganidis D., Chatzinikolaou A., Jamurtas A.Z., Carlos Barbero J., Tsoukas D., Theodorou A.S., Fatouros I. (2013). The time-frame of acute resistance exercise effects on football skill performance: The impact of exercise intensity. J. Sports Sci..

[9] Mujika I., Santisteban J., Castagna C. (2009). In-season effect of short-term sprint and power training programs on elite junior soccer players. J. Strength Cond. Res..

[10] Gorostiaga E.M., Izquierdo M., Ruesta M., Iribarren J., González-Badillo J.J., Ibáñez J. (2004). Strength training effects on physical performance and serum hormones in young soccer players. Eur. J. Appl. Physiol..

[11] Lesinski M., Prieske O., Granacher U. (2016). Effects and dose–response relationships of resistance training on physical performance in youth athletes: A systematic review and meta-analysis. Br. J. Sports Med..

[12] Brocherie F., Girard O., Forchino F., Al Haddad H., Dos Santos G.A., Millet G.P. (2014). Relationships between anthropometric measures and athletic performance, with special reference to repeated-sprint ability, in the Qatar national soccer team. J. Sports Sci..

[13] Spencer M., Bishop D., Dawson B., Goodman C. (2005). Physiological and Metabolic Responses of Repeated-Sprint Activities. Sports Med..

[14] Saidi K., Ben Abderrahman A., Boullosa D., Dupont G., Hackney A.C., Bideau B., Pavillon T., Granacher U., Zouhal H. (2020). The Interplay Between Plasma Hormonal Concentrations, Physical Fitness, Workload and Mood State Changes to Periods of Congested Match Play in Professional Soccer Players. Front. Physiol..

[15] Cherouveim E.D., Miliotis P.G., Koskolou M.D., Dipla K., Vrabas I.S., Geladas N.D. (2023). The effect of skeletal muscle oxygenation on hemodynamics, cerebral oxygenation and activation, and exercise performance during incremental exercise to exhaustion in male cyclists. Biology.

[16] Bassett D.R., Howley E.T. (2000). Limiting factors for maximum oxygen uptake and determinants of endurance performance. Med. Sci. Sports Exerc..

[17] Saidi K., Abderrahman A.B., Hackney A.C., Bideau B., Zouita S., Granacher U., Zouhal H. (2021). Hematology, hormones, inflammation, and muscle damage in elite and professional soccer players: A systematic review with implications for exercise. Sports Med..

[18] Saidi K., Zouhal H., Rhibi F., Tijani J.M., Boullosa D., Chebbi A., Hackney A.C., Granacher U., Bideau B., Ben Abderrahman A. (2019). Effects of a six-week period of congested match play on plasma volume variations, hematological parameters, training workload and physical fitness in elite soccer players. PLoS ONE.

[19] Silva A.S.R., Santhiago V., Papoti M., Gobatto C.A. (2008). Hematological parameters and anaerobic threshold in Brazilian soccer players throughout a training program. Int. J. Lab. Hematol..

[20] Borg G.A. (1982). Psychophysical bases of perceived exertion. Med. Sci. Sports Exerc..

[21] Weiner J.S., Lourie J.A. (1981). Practical Human Biology.

[22] Selmi M.A., Elloumi M., Hambli M., Sellami M., Yahmed M.H., Sassi R.H. (2016). Reliability, criterion and construct validity of multiple repeated-sprint sets test in young soccer player. Sci. Sports.

[23] Fitzsimons M., Dawson B., Ward D. (1993). Cycling and running test of repeated sprint ability. Aust. J. Sci. Med. Sport.

[24] Dill D.B., Costill D.L. (1974). Calculation of percentage changes in volumes of blood, plasma, and red cells in dehydration. J. Appl. Physiol..

[25] Cohen J. (1988). Statistical Power Analysis for the Behavioral Sciences.

[26] Kotzamanidis C., Chatzopoulos D., Michailidis C., Papaiakovou G., Patikas D. (2005). The effect of a combined high-intensity strength and speed training program on the running and jumping ability of soccer players. J. Strength Cond. Res..

[27] Adams K., O’Shea J.P., O’Shea K.L., Climstein M. (1992). The effect of six weeks of squat, plyometric and squat-plyometric training on power production. J. Strength Cond. Res..

[28] Bobbert M.F., Van Soest A.J. (1994). Effects of muscle strengthening on vertical jump height: A simulation study. Med. Sci. Sports Exerc..

[29] Hakkinen K. (1994). Neuromuscular adaptation during strength training, ageing, detraining, and immobilization. Crit. Rev. Phys. Rehabil. Med..

[30] Hoffmann T. (1999). The meanings of competency. J. Eur. Ind. Train..

[31] Fatouros I.G., Jamurtas A.Z., Leontsini D., Taxildaris K., Aggelousis N., Kostopoulos N., Buckenmeyer P. (2000). Evaluation of plyometric exercise training, weight training, and their combination on vertical jumping performance and leg strength. J. Strength Cond. Res..

[32] Meyer T., Meister S. (2011). Routine Blood Parameters in Elite Soccer Players. Int. J. Sports Med..

[33] Heisterberg M.F., Fahrenkrug J., Krustrup P., Storskov A., Kjær M., Andersen J.L. (2013). Extensive monitoring through multiple blood samples in professional soccer players. J. Strength Cond. Res..

[34] Anđelković M., Baralić I., Đorđević B., Stevuljević J.K., Radivojević N., Dikić N., Stojković M. (2015). Hematological and biochemical parameters in elite soccer players during a competitive half season. J. Med. Biochem..

[35] Brocherie F., Millet G.P., Hauser A., Steiner T., Wehrlin J.P., Rysman J., Girard O. (2015). Association of hematological variables with team-sport specific fitness performance. PLoS ONE.

